# Renin-angiotensin system activation correlates with microvascular dysfunction in a prospective cohort study of clinical sepsis

**DOI:** 10.1186/cc8887

**Published:** 2010-02-22

**Authors:** Kevin C Doerschug, Angela S Delsing, Gregory A Schmidt, Alix Ashare

**Affiliations:** 1 Department of Internal Medicine, University of Iowa Carver College of Medicine, 200 Hawkins Drive, Iowa City, Iowa, 52242, USA; 2 Department of Internal Medicine, Dartmouth Medical School, One Medical Center Drive, Lebanon NH, 03756, USA

## Abstract

**Introduction:**

Microvascular dysregulation characterized by hyporesponsive vessels and heterogeneous bloodflow is implicated in the pathogenesis of organ failure in sepsis. The renin-angiotensin system (RAS) affects the microvasculature, yet the relationships between RAS and organ injury in clinical sepsis remain unclear. We tested our hypothesis that systemic RAS mediators are associated with dysregulation of the microvasculature and with organ failure in clinical severe sepsis.

**Methods:**

We studied 30 subjects with severe sepsis, and 10 healthy control subjects. Plasma was analyzed for plasma renin activity (PRA) and angiotensin II concentration (Ang II). Using near-infrared spectroscopy, we measured the rate of increase in the oxygen saturation of thenar microvascular hemoglobin after five minutes of induced forearm ischemia. In so doing, we assessed bulk microvascular hemoglobin influx to the tissue during reactive hyperemia. We studied all subjects 24 hours after the development of organ failure. We studied a subset of 12 subjects at an additional timepoint, eight hours after recognition of organ failure (early sepsis).

**Results:**

After 24 hours of resuscitation to clinically-defined endpoints of preload and arterial pressure, Ang II and PRA were elevated in septic subjects and the degree of elevation correlated negatively with the rate of microvascular reoxygenation during reactive hyperemia. Early RAS mediators correlated with microvascular dysfunction. Early Ang II also correlated with the extent of organ failure realized during the first day of sepsis.

**Conclusions:**

RAS is activated in clinical severe sepsis. Systemic RAS mediators correlate with measures of microvascular dysregulation and with organ failure.

## Introduction

Sepsis is an inflammatory response to infection, and multiple organ failure contributes to the mortality of afflicted patients. Early restoration of systemic oxygen delivery aids in the resuscitation of patients with septic shock, but in contrast to other forms of shock, microvascular perturbations persist despite optimized global hemodynamics [1]. Because a disturbed microvasculature results in diminished nutrient extraction [2], clinicians now search for therapeutic goals of microvascular resuscitation in severe sepsis [3].

Direct imaging of the sublingual microcirculations of septic humans reveals decreased capillary density and heterogeneous flow patterns compared to controls [4]. Sepsis disrupts endothelial signaling and diminishes response to local vasodilators [5], suggesting that heterogeneous flow patterns may be due to abnormal vessel regulation. Indeed, hyperemic responses to transient ischemia are impaired in the septic human microvasculature [6-8], and the degree of impairment is associated with the degree of organ failure [9].

Angiotensin II (Ang II) is a potent vasoconstrictor and diminishes vasodilator responses in arteries [10]. In addition to direct effects on vascular tone, Ang II affects multiple aspects of microvascular function through promotion of leukostasis [11], induction of capillary permeability [12], and depletion of glutathione [13]. The renin-angiotensin system (RAS) is activated in sepsis, and recent studies implicate Ang II in the pathogenesis of acute lung injury in animal models [14]. Although RAS mediators are present in the blood and microcirculatory structures during sepsis, the relationships between RAS and microvascular function during clinical sepsis have not been investigated. We hypothesize that RAS activation is associated with impaired microvascular regulation and organ dysfunction in patients with sepsis. To test this hypothesis, we studied a prospective cohort of human subjects with severe sepsis. Circulating mediators of RAS were measured and compared to both microvascular responses during reactive hyperemia as well as to organ dysfunction.

## Materials and methods

### Study design

We studied 30 consecutive patients in our Medical Intensive Care Unit who fulfilled enrollment criteria, including 1) severe sepsis, defined as signs of systemic inflammation in the setting of probable or confirmed infection, as originally described in a consensus statement [15] and a more recently refined consensus [16], and confirmed by attending critical care physician evaluation; 2) organ failure for no more than 24 hours; 3) signed informed consent, including from surrogate decision-makers. Patients were excluded for the following reasons: 1) recent chemotherapy; 2) recent steroid or immunosuppressive agents; 3) severe peripheral vascular disease, dialysis fistulas, or mastectomies that would preclude safe forearm occlusion; 4) "Do Not Resuscitate" order *at time of enrollment*. Ten of these 30 subjects were included in a previous report that validated the NIRS methodology [9]. In addition to sepsis subjects, we studied 10 healthy volunteers that did not take any medications. This study was performed in a manner compliant with the Helsinki Declaration, and approved by the University of Iowa Institutional Review Board.

Sepsis subjects were studied 24 hours after the clinical recognition of organ dysfunction, corresponding to a time of clinical significance [17,18], and when the prognostic value of microvascular function has been well studied [4,9,19]. Twelve of these septic subjects were enrolled early such that an initial study could also be performed eight hours after the recognition of organ dysfunction; this subset of subjects was evaluated following the phase of Early Goal Directed Therapy, after which vascular resuscitation may be less effective [20]. All resuscitation goals and methods were left to the ICU team. Clinical data were collected prospectively. Organ failure was assessed using the Sequential Organ Failure Assessment (SOFA) scoring system, using the 24 hour worst-case score for each organ system as originally validated [18]. Vasoconstrictor use was classified according to criteria for the SOFA cardiovascular component. Accordingly, low dose vasoconstrictors include Dopamine > 5 mcg/kg/min or Norepinephrine ≤ 0.1 mcg/kg/min, and high-dose vasoconstrictors include Dopamine > 15 mcg/kg/min or Norepinephrine > 0.1 mcg/kg/min. Since the validation of SOFA scores, arginine vasopressin infusions have been shown to decrease the need for additional vasopressors and now are used commonly. Because vasopressin effects on blood pressure are considered similar to those of norepinephrine [21], subjects on vasopressin as a single vasoactive agent were given a cardiovascular component score of 3, while those on vasopressin plus additional agents were given a score of 4.

### Measurements of RAS activity

Blood was collected using ethylenediaminetetraacetate (EDTA)-filled vacuum phlebotomy tubes. Samples were immediately placed on ice and plasma was separated and frozen to -80°C within 30 minutes of blood draw. The rate of generation of angiotensin in ex-vivo plasma, or plasma renin activity (PRA), was assayed using a commercial radioimmune assay (RIA) kit (DiaSorin, Stillwater, MN, USA). One tube was prechilled and prefilled with the converting enzyme inhibitor bestatin to prevent ex-vivo generation of Ang II. Subsequently, the plasma concentration of Ang II was measured using a commercial RIA kit (ALPCO, Salem, NH, USA).

### Microvascular responses to reactive hyperemia

We utilized near infrared spectroscopy (NIRS) to monitor microvascular responses to reactive hyperemia in thenar skeletal muscle [9]. NIRS detects the oxygen saturation of hemoglobin specifically in skeletal muscle tissue microvasculature (S_t_O_2_) with little influence from myoglobin or from blood flow to skin or other tissues [22,23]. The Inspectra 325 Tissue Spectrometer (Hutchinson Technology, Hutchinson, MN, USA) utilizes 15 mm spacing between emission and detection points, and provides tissue attenuation measurements at four discreet wavelengths (680, 720, 760, and 800 nm) [24]. Prior to NIRS testing, patients inhaled 100% oxygen to maximize S_p_O_2_. Using techniques previously validated [9], forearm stagnant ischemia was maintained via a vascular cuff inflated to 250 mm Hg for five minutes, then the cuff was deflated rapidly. We defined the reoxygenation rate as the rate of increase of S_t_O_2 _during the immediate 14 seconds after the release of ischemia. This technique represents the summative rate of all arterial influx to the tissue microvasculature and hence the microvascular response to reactive hyperemia [9]. To determine the reproducibility of our measurements, four additional normal control subjects underwent repeated ischemia/reoxygenation testing with 10 minutes rest between ischemic periods.

Microvascular responses were evaluated immediately following phlebotomy for RAS mediators. The family of one septic subject refused stagnant ischemia after the enrollment process due to deterioration of clinical status; the previously collected clinical and plasma data are included in the analysis.

### Statistical analysis

Clinical, NIRS, and plasma data were analyzed with GraphPad Prism software v4.0 (San Diego, CA, USA). Candidate groups for comparison were assessed with a normality test, and Student's t-test was utilized if appropriate. Medians of two groups with non-Gaussian distributions were compared with Mann-Whitney tests, whereas medians of three groups with non-Gaussian distributions were compared with the Kruskal-Wallis test; post-hoc analyses of significant differences (α < 0.05) were investigated with Dunn's Multiple Comparison Test. A Pearson correlation coefficient was calculated to compare linear relationships between two continuous variables with Gaussian distributions; a Spearman coefficient was calculated when non-Gaussian distributions were noted. Individual statistical tests are specifically stated in each figure legend.

## Results

Thirty subjects fulfilled our enrollment criteria, including 12 subjects enrolled early such that an eight-hour study could also be performed. Clinical data are summarized in Table 1. Our subjects demonstrated a broad age range and a slight male predominance. Pneumonia was the most common infection leading to sepsis. Vasoconstrictor use was common, as was mechanical ventilation, while nearly half of our patients developed extensive organ dysfunction culminating in a SOFA score of 10 or greater (a predictor of 50% mortality). Patients with severe sepsis were resuscitated according to clinician preference, including a mean total fluid intake over eight liters in the first 24 hours of ICU care. The mean value of mean arterial pressures in our subjects was 69 mm Hg (SD 10.4 mm Hg). Although no subject had chronic renal failure requiring renal replacement therapy prior to enrollment, the median serum creatinine was 1.7 mg/dL. Overall, our subjects experienced 67% survival. These features represent a typical severe sepsis population at high risk of death.

**Table 1:** Clinical data of severe sepsis subjects

	Mean	Range
Age (years)	56	31 to 85
Mean arterial pressure (mm Hg)	69	48 to 91
Heart rate (beats/min)	91	51 to 124
S_p_O_2 _(%)	97	90 to 100
Hemoglobin, (g/dL) *during NIRS*	11	8.6 to 22.4
Blood Lactate*, *maximum value*	3.7	0.7 to 10.3
Serum Creatinine, (mg/dL)	2	0.5 to 7.6
SOFA Score	10	1 to 19

	***n***	**%**

Total enrolled	30	100
Male gender	17	57
Severe organ failure†	14	47
Vasoconstrictor use^‡^	21	70
Mechanical Ventilation	21	70
Survive, *in-hospital*	20	67
Source of Infection		
Pneumonia	15	50
Genitourinary	5	17
Abdominal	5	17
Endovascular^‡‡^	4	13
Multiple foci	1	3

S_p_O_2_, arterial oxygen saturation by pulse oximetry; SOFA, sequential organ failure assessment. *n = 25 subjects. ^†^Severe organ failure defined as SOFA ≥ 10. ^‡ ^Vasoconstrictor use includes dopamine > 5 mcg/kg/min or any dose of norepinephrine or vasopressin. ^‡‡^Endovascular denotes bacteremia without detectable extravascular source of infection.

**Figure 1:**
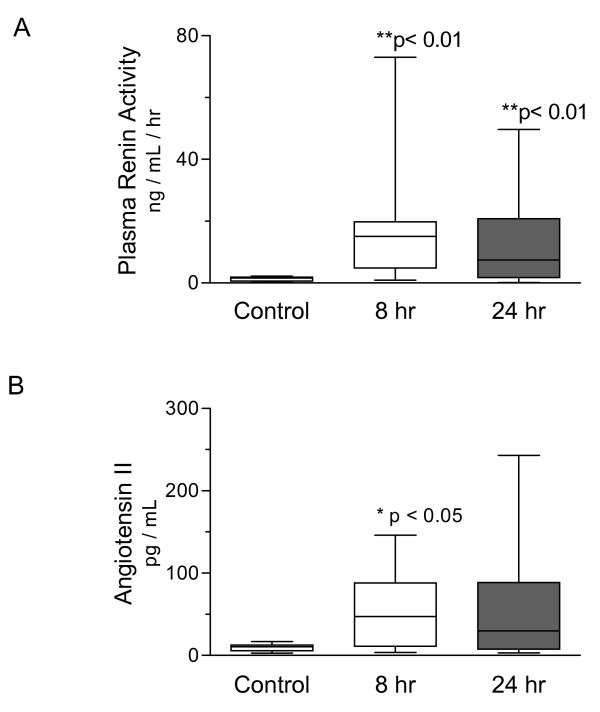
**Circulating RAS mediators are prevalent in the septic circulation**. Plasma renin activity (Panel **A**) and the plasma concentration of angiotensin II (Panel **B**) were assessed in control (n = 10) and septic subjects. At eight hours following the recognition of organ dysfunction, both PRA and Ang II were elevated in septic subjects (n = 12). Despite resuscitation to clinical endpoints, median values for PRA (7.4 ng/mL/hr, range 0.1 to 49.7 ng/mL/hr) and Ang II (29.8 pg/mL, range 3.1 to 242.8 pg/mL) remained elevated at 24 hours (n = 30). Data depict median, interquartile range, and range for each column. * *P *< 0.05, ** *P *< 0.01 compared to control, Kruskal-Wallis test, and Dunn's Multiple Comparison post-hoc test.

**Figure 2:**
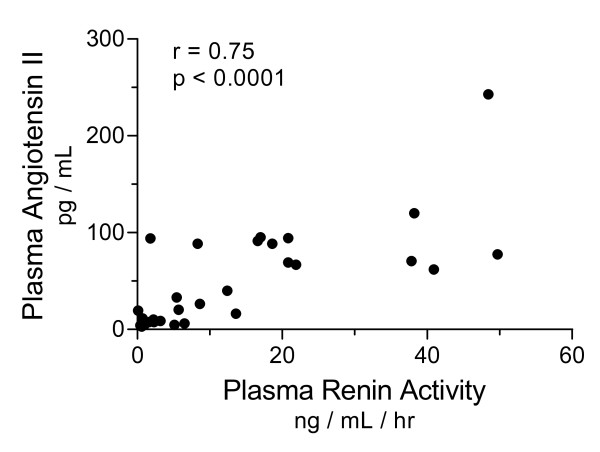
**Plasma renin activity correlates with plasma concentration of angiotensin II in septic patients**. PRA and Ang II were measured 24 hours after the recognition of organ dysfunction in 30 septic patients. Correlation analysis showed a significant relationship between these factors (Spearman r = 0.75; *P *< 0.0001).

Median values for PRA (7.4 ng/mL/h, range 0.1 to 49.7 ng/mL/h) and Ang II (29.8 pg/mL, range 3.1 to 242.8 pg/mL) were elevated at 24 hours, despite resuscitation to clinical endpoints of preload and mean arterial pressure (see Figure 1). There was no relationship between serum creatinine and either measure of RAS activation. However, PRA correlated with total SOFA score (Spearman r = 0.44, *P *= 0.01). Ang II did not correlate with SOFA scores at 24 hours. We compared values of PRA and Ang II to assess consistency within an intact biologic system and found a strong correlation between these mediators (Spearman r = 0.75; *P *< 0.0001; see Figure 2). Mean arterial pressure did not correlate with PRA (r = -0.31, *P *= 0.10) and only weakly correlated with Ang II (r = -0.43, *P *= 0.02). Since many of our subjects were being treated with vasoactive drugs, and because catecholamines may stimulate renin release, we sought interactions between such therapy and circulating RAS mediators. Concentrations of the potent vasoconstrictor Ang II were similar in subjects receiving exogenous vasoconstrictor infusions and those not receiving these drugs (mean 54.9 pg/mL, SD 56.4 vs 37.5 pg/mL, SD 41.6; normality test *P *> 0.1 for each group, Student t-test *P *= 0.4).

**Figure 3:**
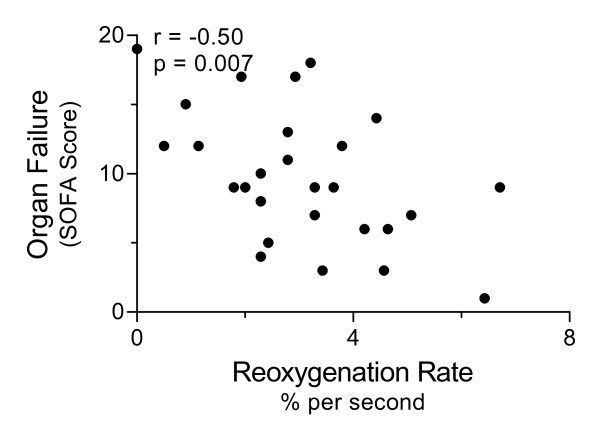
**Microvascular responses to reactive hyperemia correlate inversely with organ dysfunction in severe sepsis**. The microvascular response to reactive hyperemia was assessed by NIRS measures of thenar reoxygenation rates following induced forearm ischemia in 28 subjects. Correlation analysis showed a significant inverse relationship between microvascular reoxygenation rates and the degree of organ failure as assessed with the Sequential Organ Failure Assessment (SOFA) score (Pearson r = -0.50, *P *= 0.007).

**Figure 4:**
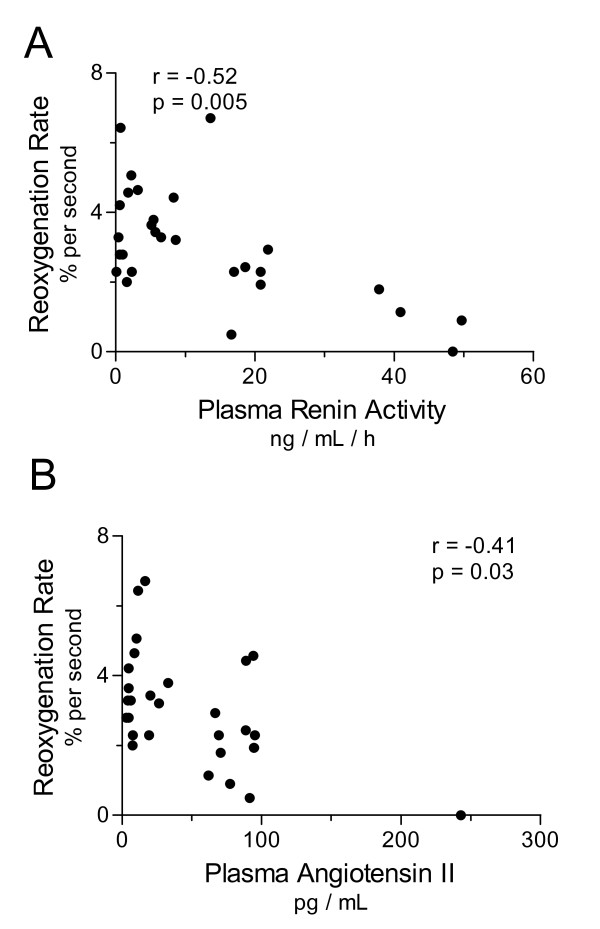
**Circulating RAS mediators correlate inversely with the microvascular responses to reactive hyperemia**. Circulating RAS mediators were assessed by radioimmune assay of plasma from septic subjects 24 hours following the clinical onset of organ dysfunction. Correlation analysis showed both plasma renin activity (Panel **A**; Spearman r = -0.52, *P *= 0.005) and plasma angiotensin II concentration (Panel **B**; Spearman r = -0.41, *P *= 0.03) had significant inverse linear relationships with thenar reoxygenation rates, or microvascular responses to reactive hyperemia.

At the same time that plasma was sampled for PRA and Ang II, we assessed the microvascular response to reactive hyperemia using NIRS. The reoxygenation rate following ischemia was impaired in septic compared to control subjects (mean 3.0% per sec (SD 1.6) vs. 4.8% per sec (SD1.1); t-test *P *= 0.003). The coefficient of variability of the reoxygenation rate in normal control subjects was 23%, similar to previous reports [25]. The reoxygenation rate correlated negatively with the degree of organ dysfunction in septic subjects (Pearson r = -0.50, *P *= 0.007; see Figure 3), confirming our prior findings [9]. The reoxygenation rate was lower in those subjects receiving exogenous vasoconstrictors (mean 2.6% per sec (SD 1.6)) than in those not on vasoconstrictors (mean 4.0% per sec (SD 1.3); t-test *P *= 0.03). This did not appear to depend on drug dose as reoxygenation rates for those on high dose vasoconstrictors (2.6% per sec, SD 1.8) were similar to those on lower doses (2.7% per sec, SD 0.78; t-test *P *= 0.88). Similarly, reoxygenation rates were lower in 20 septic subjects receiving continuous sedation during mechanical ventilation (2.45% per sec, SD 1.21) compared to septic subjects that were not ventilated (4.27% per sec, SD 1.68; t-test *P *= 0.03). Within the subset of ventilated septic subjects, reoxygenation rates still correlated with total SOFA score (r = -0.48; *P *= 0.037). A novel finding is that these microvascular responses correlated with RAS mediators in septic subjects. We found negative correlations between reoxygenation rates and both PRA (Spearman r = -0.52, *P *= 0.005) and Ang II (Spearman r = -0.41, *P *= 0.03, see Figure 4).

**Figure 5:**
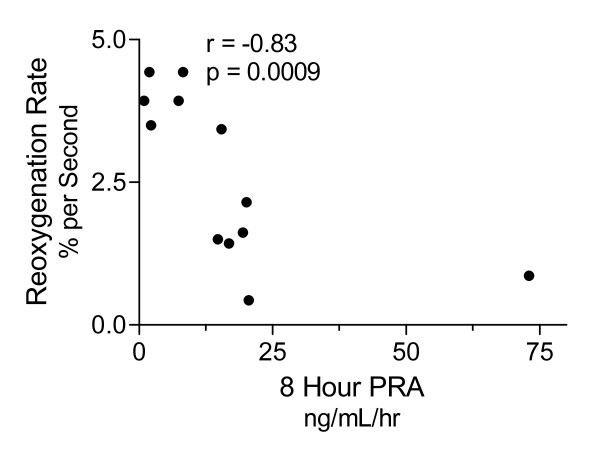
**Early RAS activation correlates with microvascular dysfunction**. Plasma renin activity was assessed by radioimmune assay of plasma from a subset of 12 subjects studied eight hours following the recognition of organ failure. Correlation analysis showed PRA had a significant inverse relationship (Spearman r = -0.83, *P *= 0.0009) with microvascular reoxygenation rates.

**Figure 6:**
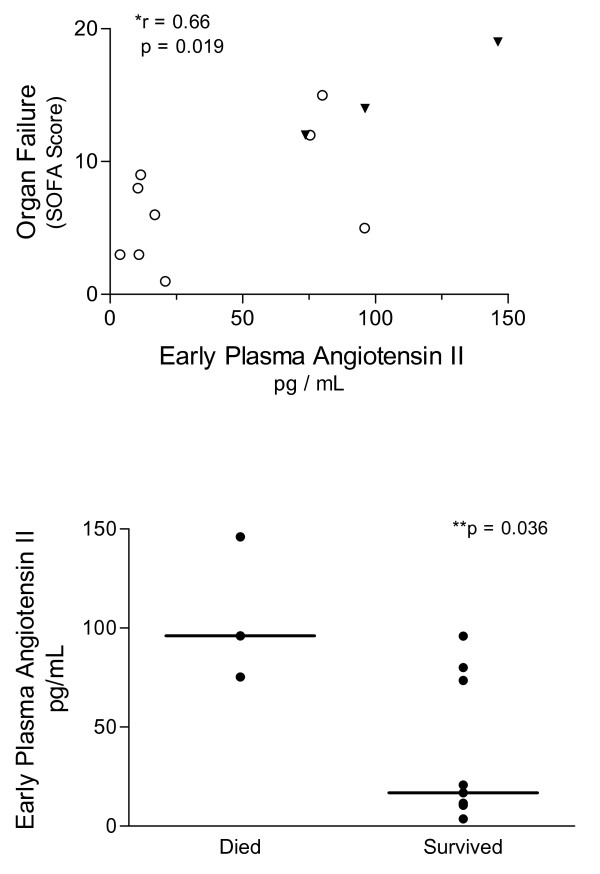
**Early plasma angiotensin II concentration correlates with organ failure in severe sepsis**. Plasma angiotensin II concentration was measured eight hours after the recognition of organ failure in 12 septic subjects. Panel **A**: Correlation analysis of these 12 subjects showed a significant relationship (Spearman r = 0.66; *P = 0.019) between Ang II and the extent of organ failure realized during the first day of ICU care as determined by the Sequential Organ Failure Assessment (SOFA) Score. Data shown includes subjects that died (black triangles) or survived hospitalization (open circles). Panel **B**: Early Ang II concentrations in those that ultimately survived hospitalization (mean 36.0 pg/mL, SD 36 pg/mL) were lower than those in subjects that died (mean 105.8 pg/mL, SD 36.4 pg/mL; ** normality test *P *> .1; Student t-test *P *= 0.016).

In the subset of 12 subjects studied eight hours following the recognition of sepsis-induced organ dysfunction, our findings were quite similar. Three subjects (25%) studied at this early timepoint ultimately did not survive hospitalization. The median PRA was significantly elevated in early septic subjects (15.1 ng/mL/h, range 0.9 to 73 ng/mL/h) compared to controls (1.5 ng/mL/h, range 0.1 to 2.2 ng/mL/h; see Figure 1, Panel A). Circulating Ang II was also increased in sepsis subjects (median 47.2 pg/mL, range 3.7 to 146 pg/mL) at this early timepoint (control median 10.6 pg/mL, range 2.8 to 17 pg/mL; see Figure 1, Panel B). Early PRA correlated negatively with microvascular reoxygenation rates measured at the same timepoint (Spearman r = -0.83, *P *= 0.0009; see Figure 5). Strikingly, the plasma concentration of Ang II early in sepsis correlated with the extent of organ dysfunction realized during the first day of ICU care (Spearman r = 0.66, *P *= 0.019; see Figure 6). In parallel, early Ang II concentrations in those that ultimately survived hospitalization (mean 36.0 pg/mL, SD 36 pg/mL) were lower than those in subjects that died (mean 105.8 pg/mL, SD 36.4 pg/mL; normality test *P *> .1; Student t-test *P *= 0.016).

## Discussion

We found that circulating mediators of RAS are prevalent in clinically severe sepsis. As such we have confirmed prior studies [26,27] and extended the evaluation of RAS mediators to two relevant timepoints during resuscitation. Additionally, we have demonstrated relationships between RAS mediators and impaired physiology within human septic subjects.

Our previous work documented that arteriolar influx to skeletal muscle tissue was most impaired in septic patients with profound vital organ failure [9]. Using similar techniques, others have found this measure to be most impaired in septic patients who do not survive [19]. The negative linear relationship between microvascular regulation and organ failure in our current study substantiates the reliability and relevance of this physiologic measurement.

Several therapeutic interventions in the care of septic subjects can potentially alter vascular responses. Continuous infusions of propofol, benzodiazepines, and opiates were used in our subjects that required mechanical ventilation, and are known to impair vasodilatory responses. That reoxygenation rates correlated with overall severity of illness score even within this subgroup suggests that sedative infusions themselves are not the major cause of impaired responses in our subjects.

It is interesting that responses to reactive hyperemia were most impaired in our subjects receiving exogenous vasoconstrictors (with a modest test of significance and with no evidence of a dose-response), while previously we found no relationship between vasoconstrictor use and diminished responses in septic subjects. Other groups have similarly described only a limited relationship between exogenous vasoconstrictors and diminished microvascular responses in septic patients [19]. When norepinephrine infusions are titrated to escalating arterial pressure targets in septic patients, some subjects have an *ideal *resuscitation point above or below which microvascular perfusion is impaired [28]. This leaves open the possibility that some of our observed microvascular dysfunction may have been due to inadequate resuscitation. However, this occurs in a minority of septic subjects whereas microvascular flow is generally not altered when norepinephrine is titrated to mean arterial pressures ranging from 60 to 90 mm Hg [29]. Catecholamines alter vasodilatory responses, but any analysis of vasomotor responses must consider that circulating endogenous vasoconstrictors are elevated in sepsis and likely affect hyperemic responses even in patients that don't receive vasoconstrictor infusions. The limited relationship between vasoconstrictor infusions and hyperemic responses in our studies suggest that exogenous catecholamines do not play a large role (compared to endogenous factors) in dampening hyperemic responses. Because Ang II was equally elevated in patients who did or did not receive exogenous vasoconstrictors, we are urged to investigate relationships between circulating RAS mediators and microvascular function in sepsis.

We considered that RAS activation might simply reflect glomerular hypoperfusion due to hypovolemia, hypotension, or insufficient resuscitation. The clinical use of vasopressors, mechanical ventilation, and fluid resuscitation in our subjects was consistent with aggressive resuscitative efforts during the first day of sepsis, although we did not standardize resuscitation to measures of cardiac output, pulmonary artery occlusion (wedge) pressure, or pulse pressure variation in accord with uncertainties regarding what these goals should be [30-32]. Similarly, preexisting hypertension, diabetes, and coronary disease are associated with increased RAS activity, and no doubt are co-morbid conditions in clinical sepsis. We note that the levels of PRA and Ang II measured in our septic subjects are elevated nearly two-fold compared to outpatients with risk factors for vascular disease [33,34], arguing that the acute septic state contributes to RAS activation. Although we did identify a relationship between arterial hypotension and circulating Ang II after the first day of severe sepsis, the modest statistical significance and lack of a similar relationship between hypotension and PRA (a biologic precursor to Ang II) temper our enthusiasm to declare arterial pressure a dominant factor leading to persistent RAS activation during sepsis.

Our most novel finding is the association of circulating mediators of RAS with impaired hyperemic responses to ischemia during sepsis. This association raises the possibility that sepsis stimulates RAS, which contributes to microvascular perfusion heterogeneity (manifested as impaired response to local ischemia), and that perfusion heterogeneity contributes to organ failure. We cautiously note that our studies do not define a causal role of RAS in the pathogenesis of septic microvascular dysfunction, and RAS activation may be unrelated or even compensatory for microvascular dysfunction. However, findings of increased small vessel density and decreased heterogeneity following vasodilator administration to septic subjects [35,36] suggest that an enhanced vasoconstrictor tone contributes to perturbations of the microvasculature. Thus our findings suggest that RAS contributes to the enhanced microvascular tone in human sepsis.

Ang II inhibits endothelium-dependent relaxation of resistance arteries [37] and thus modulates the response to ischemia. Antagonism of the angiotensin type 1 receptor increases blood flow to ischemic mesenteries [38] and attenuates mucosal permeability and bacterial translocation [39] in animal models of shock. In addition to direct effects on vascular tone, Ang II induces adhesion marker expression on both leukocytes and endothelial cells [40,41] and thus may propagate the hemostatic and inflammatory interactions implicated in microvascular perturbations and organ failure during sepsis. We note that early Ang II correlates with the extent of organ failure achieved during the first day, but Ang II values later in the course of sepsis do not correlate with SOFA scores. The explanation for this discrepancy is not clear. It is possible that Ang II is an early mediator in a cascade of events that results in organ failure over the first day, and as such the late concentration of Ang II is less relevant to organ failure.

Circulating precursors to Ang II also have biologic importance. It is worth noting that PRA also correlated with impaired hyperemic responses as well as SOFA scores in our studies. Inhibition of angiotensin converting enzyme (ACE) with enalapril improves endothelium-dependent relaxation in endotoxemic animals [42]. ACE inhibition decreases endothelial-derived adhesion molecules and vasoconstrictors, improves gut perfusion, and reduces organ failure in critically ill patients [26,43]. Our studies provide evidence of associations between RAS and relevant microvascular perturbations in sepsis. Importantly, our studies provide an impetus to determine if pharmacologic RAS blockade can increase microvascular function and improve septic patient outcomes.

## Conclusions

RAS mediators are present in the systemic circulation in human sepsis. Plasma renin activity and angiotensin II concentrations correlate with impairments in microvascular dysfunction, organ failure, and mortality. These derangements appear early and persist through the first day of severe sepsis despite macrovascular resuscitation.

## Key messages

▪ The renin-angiotension system (RAS) activation correlates with organ injury and mortality in clinical sepsis.

▪ Systemic RAS mediators persist in many septic patients despite macrovascular resuscitation.

▪ Microvascular responses to ischemia are impaired in clinical sepsis and correlate with vital organ function.

▪ Systemic RAS mediators correlate inversely with microvascular responses to ischemia.

▪ Future work can determine if RAS antagonism can improve microvascular function and vital organ function in clinical sepsis.

## Abbreviations

ACE: Angiotensin converting enzyme; Ang II: plasma concentration of angiotensin II; EDTA: ethylenediaminetetraacetate; NIRS: near infrared spectroscopy; PRA: plasma renin activity; RAS: Renin-Angiotensin System; RIA: radioimmune assay; SOFA: Sequential Organ Failure Assessment score; S_p_O_2_: percent oxygen saturation of arterial hemoglobin: as measured with pulse oximetry; S_t_O_2_: percent oxygen saturation of microvascular (tissue) hemoglobin: as measured with NIRS.

## Competing interests

The authors declare that they have no competing interests.

## Authors' contributions

KCD participated in subject recruitment, microvascular analysis, data analysis, and manuscript preparation. ASD participated in subject recruitment, microvascular analysis, and data analysis. GAS participated in manuscript preparation and editing. AA participated in data analysis and manuscript preparation.
